# Intrabody-based FRET probe to visualize endogenous histone acetylation

**DOI:** 10.1038/s41598-019-46573-2

**Published:** 2019-07-15

**Authors:** Chan-I Chung, Yuko Sato, Yuki Ohmuro-Matsuyama, Shinichi Machida, Hitoshi Kurumizaka, Hiroshi Kimura, Hiroshi Ueda

**Affiliations:** 1 Laboratory for Chemistry and Life Science, Institute of Innovative Research, Tokyo Institute of Technology, 4259 Nagatsuta-cho, Midori-ku, Yokohama, Kanagawa 226-8503 Japan; 2 Cell Biology Center, Institute of Innovative Research, Tokyo Institute of Technology, 4259 Nagatsuta-cho, Midori-ku, Yokohama, Kanagawa 226-8503 Japan; 3 Laboratory of Structural Biology, Graduate School of Advanced Science & Engineering, Waseda University, 2-2 Wakamatsu-cho, Shinjuku-ku, Tokyo, 162-8480 Japan; 4 Present Address: Institute for Quantitative Biosciences, The University of Tokyo, 1-1-1, Yayoi, Bunkyo-ku, Tokyo, 113-0032 Japan; 5 Present Address: Department of Pharmaceutical Chemistry, University of California San Francisco, 555 Mission Bay Blvd South, San Francisco, 94158 California USA

**Keywords:** Fluorescent proteins, Acetylation, Chemical modification

## Abstract

Post-translational histone modifications are major regulators of gene expression. However, conventional immunoassays do not provide sufficient information regarding their spatial and temporal dynamic changes. Fluorescence/Förster resonance energy transfer (FRET)-based probes are capable of monitoring the dynamic changes associated with histone modifications in real-time by measuring the balance between histone-modifying enzyme activities. Recently, a genetically encoded histone-modification fluorescent probe using a single-chain variable region (scFv) fragment of a specific antibody was developed. The probe, modification-specific intracellular antibody, is capable of monitoring histone-acetylation levels in both cultured cells and living organisms based on the ratio of fluorescence intensities between the cell nucleus and cytoplasm. In this study, we constructed a FRET probe composed of yellow fluorescent protein attached at the N-terminus of an acetyl H3K9-specific scFv, tethered to a cyan fluorescent protein. When the FRET probe was expressed in human cells, both FRET efficiency and fluorescence intensity in the nucleus increased following histone-deacetylase inhibitor treatment. Using these two parameters, endogenous histone-acetylation levels were quantified over a high dynamic range. This probe provides a simple approach to quantify spatial and temporal dynamic changes in histone acetylation.

## Introduction

Histones are relatively small proteins that associate with and help package DNA into chromatin in the nucleus. Since the correlation between histone-acetylation levels and RNA-synthesis activity was first described by V. Allfrey^1^, many different histone post-translational modifications (PTMs) have been reported. These modifications regulate chromatin structure and recruit remodeling enzymes. Among these modifications, histone acetylation is highly dynamic and regulated by histone acetyltransferases (HATs) and histone deacetylases (HDACs). Histone acetylation results in nucleosome unfolding, thereby increasing access to DNA by transcription factors^2^. HDACs remove acetyl groups from lysine residues and stabilize local chromatin architecture, consistent with the predominant role of HDACs as transcriptional repressors.

Recently, histone-modification patterns have been considered as a biomarker of tumor stage. Histone acetylation is often associated with the activation of tumor-promoting genes, while histone deacetylation may result in genomic instability and increased proliferation due to silencing of tumor-suppressor genes. For example, levels of both H3K4me2 and H3K9ac are low in normal tissue^3^, while low H3K4me2 levels with high H3K9ac levels are observed in lung cancer^4^. Global levels of H3K9ac and H3K56ac that decrease in response to DNA damage may reflect an effect of transcriptional inhibition^5^. H3K9ac is also considered as an epigenetic marker associated with transcriptionally active chromatin during human embryonic stem-cell differentiation. Western blot and chromatin immunoprecipitation (ChIP) analysis revealed that differentiation results in a global reduction of H3K9 acetylation in human embryonic stem cells^6^.

Conventional methods that utilize specific antibodies, including immunoblotting, enzyme-linked immunosorbent assays, and ChIP, are widely used for the analysis of histone acetylation. While capable of measuring modification levels in fixed or extracted samples, these methods provide minimal information concerning temporal and spatial dynamic changes to histones in living cells and organisms. To this end, several genetically encoded fluorescence resonance energy transfer (FRET)-based probes^7–9^ and bioluminescence-based probes^10^ have been developed to monitor the dynamic changes of modification levels in cells. The general design of FRET- or bioluminescence-based probes involves fusing a substrate amino-acid sequence to histone-modifying enzymes with a modification-binding domain as a sensor, and also attaching either a FRET fluorescent-protein pair or luciferase fragments to the N- and C-termini as reporters. The association of modification-binding domains to modified-substrate domains triggers conformational changes in FRET-based probes or reconstitution of bioluminescent proteins, with either result generating measurable signals. However, the dissociation constants of modification-binding proteins are 1–200 μM for chromodomains^11^ and 3–300 μM for bromodomains^12^, which means that they bind rather weakly. Additionally, many modification-binding proteins are not specific to a single modification and could bind several modification sites.

To monitor endogenous modifications in living cells and embryos, fluorescence-labeled antigen-binding fragments from specific antibodies have been used^13^. In this system, the labeled fragment binds to its epitope of endogenous histone, with its distribution capable of changing in response to the modification level. Later, a longer-lived genetically encoded fluorescent probe, *m*odification-specific *int*racellular anti*body* (mintbody), was developed^14^. This probe was composed of a single-chain variable-region (scFv) fragment capable of being functionally expressed in the reductive cellular environment (intrabody) and tethered to an enhanced green fluorescent protein (EGFP) fused to its C-terminus. This probe retained high specificity for H3K9 acetylation and was successful in monitoring histone-acetylation levels in cultured cells and living organisms by tracking the nuclear:cytoplasmic intensity ratio of EGFP.

Here, we demonstrated that genetically encoded FRET probes using the intrabody as a sensor and a FRET fluorescent-protein pair as reporters can monitor histone-modification levels by ratiometric FRET quantification in living cells. Similar to mintbody, the probe associated with endogenous acetylated-histone tails and localized to the nucleus. After challenge with histone-deacetylase inhibitor, both FRET efficiency and nuclear-fluorescence intensity increased in a time-dependent manner. Using these two parameters, endogenous histone-acetylation levels were capable of being quantified with a high dynamic range.

## Results and Discussion

### Construction of H3K9 acetylation FRET probes

Genetically encoded FRET probes that utilize fluorescent proteins are widely used to monitor biological phenomena, including biomolecular modifications. Most FRET-based probes for cellular imaging are single polypeptides composed of a sensory domain inserted between a donor fluorescent protein (FP) and an acceptor FP^15^. Sensitized emission, also called two-color ratio imaging, is the simplest method for FRET imaging^16^. The donor is excited by a light of specific wavelength and the signals are collected using emission filters chosen based on donor and acceptor fluorescence. The dynamic changes in the FRET efficiency of intracellular probes are monitored as the fluorescence-intensity ratio of the acceptor to the donor. In this study, we developed a single-chain fusion protein consisting of two differently colored fluorescent proteins and an intrabody that specifically associates with acetylated histone H3K9 as a probe^17^.

**Figure 1:**
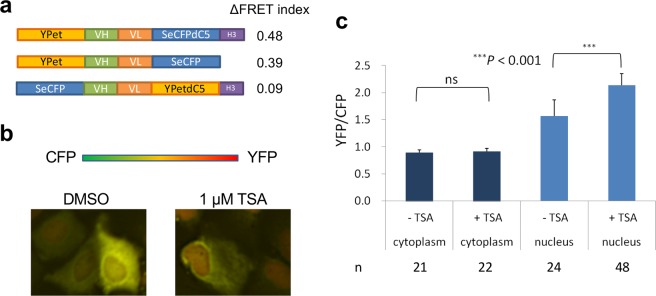
Design of intrabody-based histone acetylation FRET probes. (**a**) Schematic presentation and performance of the intrabody-based FRET probe. YPet: FRET-optimized YFP; SeCFPdC5: enhanced CFP with 5 amino acid deletion in C-terminal end; VH and VL: ScFv(19E5) intrabody for H3K9ac, H3: ARTKQTARKSTGGKAPRKQL, where K9 is underlined. FRET index is defined as the fluorescence intensity ratio of acceptor to donor, which should reflect FRET efficiency. ∆FRET index, which represents the performance of the probe, is the difference of FRET indices between those in the transiently probe-transfected U2OS cells treated with TSA (1 µM) and with dimethylsulfoxide (DMSO). (**b**) Pseudocolored FRET image of probe-transfected U2OS cells. (**c**) Ratiometric FRET quantification of fixed cells. The average with a standard deviation is indicated (*p* ~ 0.05 in cytoplasm, *p* = 4.3 × 10^−10^ in nucleus).

We first constructed potential FRET probes using red fluorescent proteins (RFPs), such as mRuby and mStrawberry, to minimize the excitation of acceptor FP by the donor excitation (Supplementary Fig. S1a). However, the response (ΔFRET index) to histone-deacetylase inhibitor, Trichostatin A (TSA) was not evident, likely due to the lower folding efficiency of those FPs. In order to improve the response, we constructed FRET probes using a cyan-yellow fluorescent protein (CFP-YFP) pair (Supplementary Fig. S1b). First, YFP (YPet) was fused at the N-terminus of anti-H3K9ac scFv 19E5 (derived of CMA310^14^) and tethered to super-enhanced cyan fluorescent protein (SeCFP), the C-terminus of which had five amino acids removed and replaced with a histone-H3 tail (Fig. 1a). Following transient probe expression in human osteosarcoma U2OS cells, the pseudocolored FRET image clearly showed that FRET efficiency was lower in the cytoplasm relative to the nucleus, even without treatment with a histone-deacetylase inhibitor, Trichostatin A (TSA) (Fig. 1b). The FRET index obtained by the ratio of YFP to CFP is summarized in Fig. 1c. The FRET index in the nucleus was higher than that observed in the cytoplasm and increased significantly following TSA treatment (*p* < 0.001), while that in the cytoplasm remained unchanged. This result indicates that the FRET index of the probe reflects the acetylation level in cells. Although NLS is not added in the probe, we could see its import into the nucleus, probably by passive diffusion across nuclear pores. We think that the mechanism is similar to that of previous mintbody construct (scFv-GFP), wherein the linkage between scFv and GFP is flexible and easy to change its shape, allowing its efficient nuclear transport.

### Effect of the H3-tail substrate at the C-terminus of the probe

To investigate whether the probe works by intramolecular binding of the scFv moiety to its own H3 tail, resulting in conformational and FRET-efficiency changes, a FRET probe without a C-terminal H3 tail was also constructed and the results were compared with those from the original probe (Fig. 1a). To our surprise, the FRET response of the probe lacking the H3 tail was similar to that of the original probe containing the tail [*p* = 0.054; (Supplementary Fig. S2)]; The FRET index in the nucleus increased following TSA treatment, although it was slightly lower than that of the original probe.

The fact that the absence of the H3 tail in the probe did not greatly affect FRET response suggested that the probe binds to the endogenous acetylated histone H3K9 rather than to the H3 sequence on the probe to increase its FRET efficiency. To further investigate the role of H3 tail in the probe, firstly, acetylation levels of the H3 tail were investigated by western blotting with anti-H3K9ac antibody using the U2OS cells expressing the probes treated with or without TSA. The acetylation levels of endogenous H3 at ~16 kDa increased by TSA treatment (Supplementary Fig. S3a, lanes 2 and 4). However, no signal was observed at ~90 kDa even after TSA treatment, suggesting low acetylation of the probe even in the presence of the C-terminal H3 tail.

Secondly, probe stability and expression level were monitored by the western blotting using anti-GFP antibody. The main bands observed at ~90 kDa identified the full-length probes, although some smaller fragments were also observed, especially for the probe without the C-terminal H3 tail (Supplementary Fig. S3b). TSA treatment did not affect the amount of both probes. Therefore, the changes in acceptor fluorescence intensity were the result of alterations in FRET efficiency and not from the expression level. From the comparison of the amounts of the full-length probe and the degraded fragments, the probe with H3 tail is considered more stable than the probe without it.

Thirdly, to test the possibility that the clustering of the probes to endogenous acetylated H3 alone is enough to explain the FRET change, another probe wherein the locations of YPet and SeCFP were exchanged to vice-versa was made (Fig. 1a). On the contrary to our expectation, this probe did not show significant TSA-dependent FRET response, suggesting that some conformational change of YPet-scFv(19E3)-SeCFP probes upon endogenous histone binding resulted in the FRET efficiency increase. This assumption was supported by the result of another construct with larger SeCFP C-terminal deletion, showing significantly low FRET response (Ypet-scFv-SeCFPdC10-H3, Supplementary Fig. S1).

Collectively, we concluded that the increases in the FRET index resulted from the conformational change of the YPet-scFv(19E3)-SeCFP probes bound to the endogenous acetylated H3 tails of histones, rather than that triggered by the binding of intrabody moieties to the H3 tails added to the C-terminus. The C-terminal H3 tail however appear to increase the stability of probe (Supplementary Fig. S3b), thereby improved the response.

### The FRET probe with different scFv specificity did not detect H3K9ac

To investigate the specificity of YPet-scFv-SeCFPdC5H3 probe, the scFv moiety of the probe was replaced with that of anti-H4K20 monomethylation antibody (15F11). After transfection of U2OS cells and treatment with 1 µM TSA as before, no significant FRET response was observed (Supplementary Fig. S4). In addition, we also made a mutant that harbors Tyr to Gly substitution at amino acid 105 in the epitope binding region (Y105G). When expressed in U2OS cells as a mintbody probe, the Y105G mutant showed considerably reduced nuclear localization (Supplementary Fig. S5). When expressed in HeLa cells as a FRET probe, it also showed reduced FRET signal (Supplementary Fig. S6). These results supported the crucial importance of H3K9ac biding activity in the scFv in the FRET probe.

### Time-lapse quantification of live U2OS cells transiently transfected with the FRET probe

**Figure 2:**
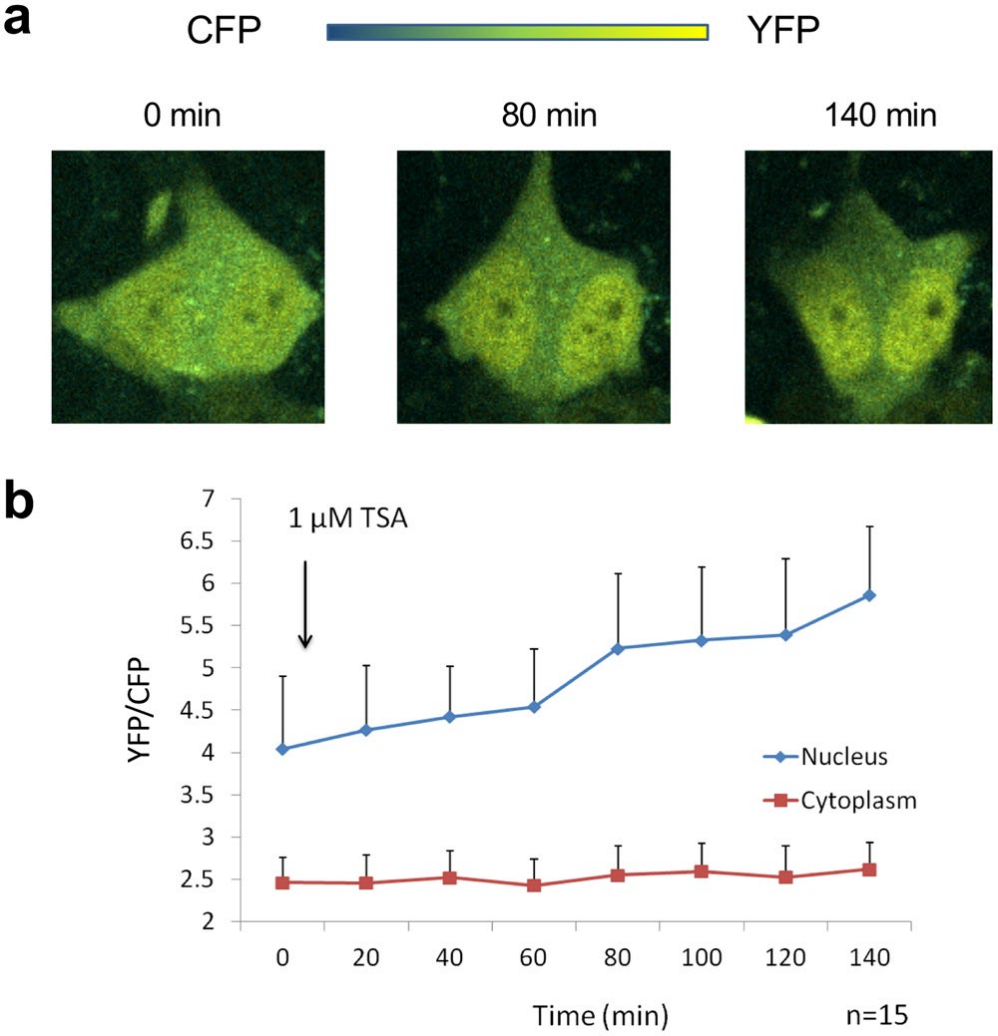
Time-lapse FRET visualization of H3K9ac in live U2OS cells. (**a**) FRET images with 405 nm excitation. After incubation with 1 μM TSA, accumulation of probe in the nucleus and increase in FRET efficiency can be observed clearly. (**b**) Average of FRET index in time-lapse quantification: ratio of 520 nm F.I. (CFP) to 477 nm F.I. (YFP) with 405 nm excitation in nucleus (blue) and in cytoplasm (red). The FRET index in the cytoplasm remained steady while that in the nucleus rose because H3K9 acetylation level increased.

To investigate whether the YPet-scFv(19E5)-SeCFPdC5H3 probe can be used to monitor the dynamic changes of histone acetylation in living cells, fluorescence images of transiently probe-transfected U2OS cells at emission wavelengths of 447 nm and 520 nm with an excitation wavelength of 405 nm were collected every 10 minutes. After the addition of 1 μM TSA, FRET probes accumulated in the nucleus and the FRET index (520 nm/447 nm ratio) increased (Fig. 2a and SI Supplementary Videos). The FRET indices of fifteen cells were quantified and the average time-lapse FRET index was plotted in Fig. 2b. The FRET index in the cytoplasm remained constant at ~2.5, even after the addition of TSA. In contrast, the FRET index in the nucleus increased from 4.0 to 5.9, reflecting increased H3K9 acetylation levels. This result demonstrates that intrabody-based FRET probes can monitor histone acetylation in living U2OS cells.

### Time-lapse quantification of HeLa cells stably transfected with the FRET probe

**Figure 3:**
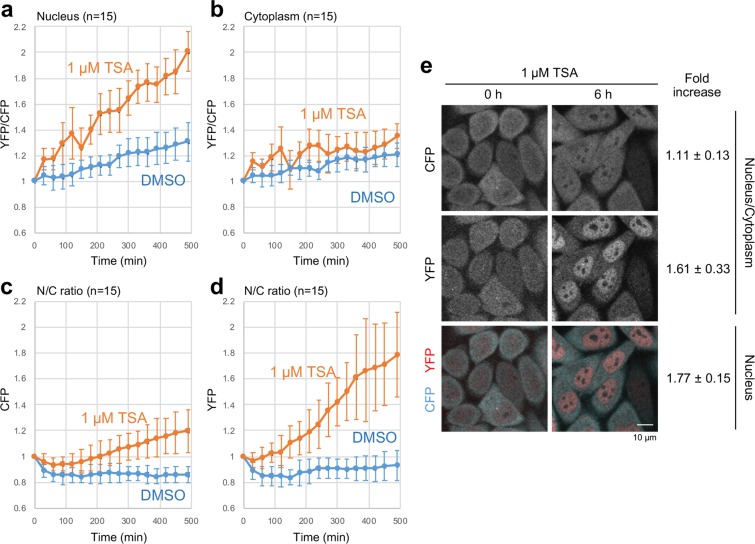
Time-lapse FRET visualization of H3K9ac in HeLa cells using YPet-scFv-SeCFPdC5H3 probe. Cells were treated with DMSO (vehicle) or 1 μM TSA were imaged every 30 min for 8 h, and the YFP/CFP ratio (**a**,**b**) or nucleus/cytoplasm ratio (**c**,**d**), relative to the value at time 0, was plotted (averages of 15 cells with the standard deviations). (**a**,**b**) Changes of FRET index (YFP/CFP), ratio of 520 nm fluorescence intensity (YFP) to 477 nm intensity (CFP) with 458 nm excitation, in the nucleus (**a**) and cytoplasm (**b**) in cells treated with DMSO (blue) and TSA (red). The FRET index increased drastically in the nucleus, but not in the cytoplasm, in TSA-treated cells. (c and d) Changes of nucleus to cytoplasmic (N/C) ratio. N/C ratio of YFP (**d**) is more increased than CFP (**c**) in TSA-treated cells. (**e**) Example images and the summary of fold change. CFP, YFP, and merged images at 0 and 6 h after the addition of TSA are shown (left). The numbers of fold increase in nucleus/cytoplasm ratio and YFP/CFP ratio at 6 h (relative to those at 0 h) are shown with the standard deviations (N = 15).

To clarify the relative merit of using the FRET probe rather than conventional mintbody to detect H3K9 acetylation, human HeLa cells were transfected with a PiggyBac expression vector either encoding YPet-scFv-SeCFPdC5H3 probe or mintbody (scFv-Venus). Afterwards, stable transfectants were selected and cloned to ensure similar expression level of the two probes. These cells were used for the time-lapse fluorescence imaging for 8 h in 1 µM TSA or DMSO (vehicle) alone (Fig. 3). Relative FRET indices (YFP/CFP fluorescence intensity ratios), as well as the nuclear/cytoplasmic intensity ratio of YFP, were increased in the nucleus in cells treated with 1 µM TSA, while mild increase was also observed for DMSO-treated cells, which probably represents the global increase of acetylation levels associated with the cell cycle progression^18^. In contrast, little increase in FRET index was observed in the cytoplasm of both TSA and DMSO-treated cells.

To confirm if the observed changes were not resulting from TSA-dependent change of the protein amounts of H3 or the FRET probe, western blot analysis was performed for the cells treated with DMSO vehicle or 1 µM TSA treatments. As a result, no TSA-dependent increase in H3 band density but that of H3K9Ac was observed, while no observable change in FRET probe amount was observed (Supplementary Fig. S7). These data indicated that the FRET index in the nucleus can be a good marker to measure the relative H3K9 acetylation levels in living cells.

**Figure 4:**
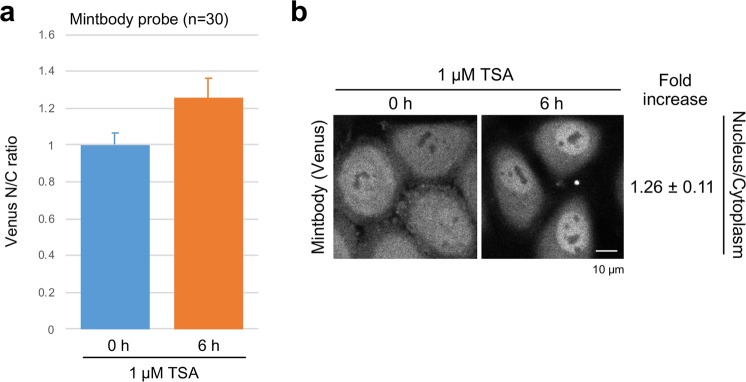
Time-lapse visualization of H3K9ac levels in HeLa cells using a mintbody. Confocal images of HeLa cells stably expressing Venus-based H3K9ac-mintbody were acquired at 0 and 6 h after addition of 1 μM TSA. The relative nucleus/cytoplasmic ratio (averages from 30 cells with the standard deviations (**a**) and representative images (**b**) are shown.

In a previous work, the nucleus/cytoplasm ratio of H3K9ac-specific mintbody was used to measure the changes in acetylation levels in U2OS cells [14]. We here measured the mintbody fluorescence intensity in stably expressing HeLa cells before and 6 h after TSA addition (Fig. 4). The nucleus/cytoplasm ratio increased modestly to 1.26 ± 0.11 fold, which is lower than the relative increase of YFP nucleus/cytoplasm ratio (1.61 ± 0.33) or the FRET index (1.77 ± 0.15) observed for the FRET probe developed here (Fig. 3e). These results clearly indicate the superior response of FRET probe over the conventional mintbody.

### Observation of FRET change *in vitro*

**Figure 5:**
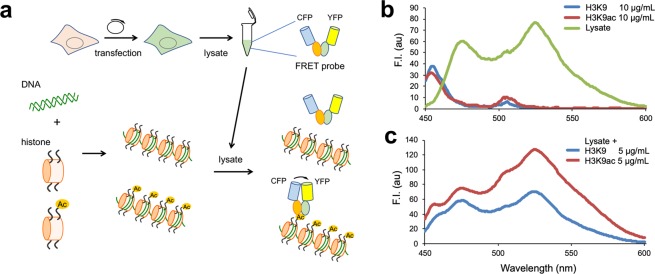
*In vitro* spectral change upon H3K9ac-containing polynucleosome. (**a**) Scheme of the assay. (**b**) Fluorescence spectrum of each component. The probe-expressing COS-7 cell lysate showed two peaks derived of SeCFP and YPet, while H3K9- and H3K9ac-containing polynucleosome showed lower peaks. (**c**) The lysate was added with 5 µg/mL of polynucleosome and measured for the spectra, which are normalized at the emission at 475 nm. Excitation wavelength was set at 430 nm.

To further ensure that the observed FRET efficiency change is due to specific recognition and binding of the probe to endogenous acetylated histone H3K9, an *in vitro* experiment using reconstituted polynucleosome and cellular lysate was performed. COS-7 cells were transfected with YPet-scFv(19E5)-SeCFPdC5H3 probe expression vector, and incubated for 72 h to allow the probe expression, before preparing cellular lysate. To such a lysate containing the FRET probe, polynucleosomes reconstituted with K9-acetylated- or non-acetylated H3 were prepared (Supplementary Fig. S8) and added at 5 µg/mL, and the fluorescence spectra measured. As shown in Fig. 5, the lysate alone exhibited the fluorescence spectrum with peaks at 475 and 525 nm, which likely represented the emission maxima of CFP and YFP, respectively. The 525 nm peak was relatively increased, when H3K9ac-containing polynucleosomes were added. The addition of polynucleosomes with non-acetylated H3 showed little spectral change, supporting the view that the probe specifically binds to acetylated H3 in nucleosomes, by which a conformational change is induced to enhance FRET efficiency.

### Development and importance of intrabody-based FRET probes

Genetically encoded FRET probes composed of sensory domains inserted between the fluorescence donor and acceptor are widely used for cell imaging. Although antibodies are powerful sensors, they are usually not amenable to intracellular detection due to the reducing milieu. The use of intrabodies that retain their structure and antigen-binding activity is a vital option for the detection of biomolecules without known natural-sensing domains or that require higher affinity and specificity for detection. Recently, a cellular oncoprotein, gankyrin, was detected by FRET using fluorescent protein-fused scFv^19^. A key difference between that study and ours is that two scFvs binding to different epitopes were used to perform sandwich detection. In their case, EGFP- and RFP-fused scFvs formed a ternary complex with gankyrin, enabling the complex to be monitored by fluorescence lifetime imaging-FRET microscopy. Although such sandwich assays might be suitable for some larger biomolecules, an assay that uses multiple probes will require careful tuning of the detection conditions in order to reproduce results.

Prior to designing an intrabody-based FRET probe for H3K9ac, we hypothesized that the association of the probe to the substrate peptide within the probe would trigger conformational changes and generate measurable FRET-efficiency changes. Although the observed FRET-index change was likely attributed to endogenous antigen-induced conformational change of the probe, our probe demonstrated that it was possible to detect PTMs by a single-chain intrabody-based FRET probe. To exploit the potential of intrabody-based FRET probes, antigen-dependent interactions between antibody-variable region fragments (V_H_ and V_L_), called the open-sandwich principle^20^, or antigen-dependent disruption of FP dimers tethered at the N-termini of V_H_/ V_L_, called an open-flower immunoassay^21^, constituted the feasible strategies. To date, hen-egg lysozyme^22^, protein-tyrosine phosphorylation^23^, and serum albumins were successfully detected by FRET-efficiency changes between FPs fused with V region fragments *in vitro*. In order to develop intrabody-based FRET probes for small molecules and for other protein-modifications, these strategies are worth pursuing in future.

## Conclusions

Here, we developed a genetically encoded antibody-based FRET probe capable of visualizing changes in histone acetylation in living cells. The FRET response was affected by the relative position of fluorescent protein pairs in the probe. Similar to a mintbody, the new probe “FRET mintbody” was capable of associating with acetylated histones and localized to the nucleus, enabling endogenous histone acetylation levels to be quantified based on FRET efficiency and probe distribution. While attachment of an H3 tail to the C-terminal region of the probe improved response by stabilizing the fusion protein, probe clustering may constitute the primary FRET-enhancement mechanism. Using the parameters of fluorescence intensity and FRET efficiency, endogenous histone acetylation levels were quantified with higher dynamic ranges compared to the previous FRET probes^7–9^ and mintbody^14^. Our probe provides a powerful approach to studying the relationships between specific histone modifications and various biological processes in living cells.

## Methods

### Construction of FRET probe expression vectors

The cDNA for super-enhanced cyan fluorescent protein (SeCFP) encoded by human-optimized codons was amplified from pPBbsr2-3594nls plasmid^24^ using specific BamSeCFP(opi)back and SeCFP(opi)XbaFor primers. The primers used in this study is summarized in Table 1. The SeCFP fragment was digested with *Bam*HI and *Xba*I and inserted into a pEGFP-N2-19E5scFv vector^14^, resulting in pN2-scFv(19E5)-SeCFP. The H3 peptide was obtained by overlap-extension polymerase chain reaction (PCR) using the H3back and H3C back primers, resulting in a 72-bp DNA fragment. SeCFP lacking five of its C-terminal residues was amplified from pPBbsr2-3594nls using the primers BamSeCFP(opi)back and SeCFP(opi)dC5H3NFor. The H3 substrate peptide was fused to the C-terminal end of SeCFP by splice-overlap PCR using the primers BamSeCFP(opi)back and H3C-XbaFor. The digested SeCFPdC5H3 fragment was inserted into the pEGFP-N2-19E5scFv vector, resulting in pN2-scFv(19E5)-SeCFPdC5H3. The cDNA for YPet encoded by human-optimized codons was amplified from the pPBbsr2-3594nls vector using the primers BglEcoRYpet(opi)Back and Ypet(opi)XhoIG3S1SacIFor. The YPet fragment was digested with *Bgl*II and *Sac*I and inserted into either the pN2-scFv(19E5)-SeCFP or pN2-scFv(19E5)-SeCFPdC5H3, resulting in pN2-YPet-scFv(19E5)-SeCFP, or pN2-YPet-scFv(19E5)-SeCFPdC5H3, respectively.

**Table 1:** Nucleotide sequences of the primers used in this study.

Primer name	Nucleotide sequence (5′–3′)
BamSeCFP(opi)back	GGAGATGGATCCATATGGTGAGCAAGGGCGAGGA
SeCFP(opi)XbaFor	CTTTGCTCATTCTAGATTAGCGGCCCAGCTCGTCCATGC
H3Cback	CGTACTAAACAGACAGC
H3back	ACAGCTCGGAAATCCACCGGCGGTAAAGCGCCACGCAAGCAG
SeCFP(opi)dC5H3NFor	GCTGTCTGTTTAGTACGAGCCATGCCGAGAGTGATCCC
SeCFP(opi)XbaFor	CTTTGCTCATTCTAGATTAGCGGCCCAGCTCGTCCATGC
H3C-XbaFor	GATCCGCCTCTAGATTACTGCTTGCGTGGCGCTT
BglEcoRYpet(opi)Back	TCATTTAGATCTGAATTCACCATGGTGAGCAAGGGC
Ypet(opi)XhoIG3S1SacIFor	CAATGGAGCTCGTGAACCGCCACCCTTCTCGAGGTACAGCTC
Y105G-for	AACGGGGGATGGTGGTTCTGACTACTGG
Y105G-rev	CCAGTAGTCAGAACCACCATCCCCCGTTC

To introduce mutations in the epitope binding region of scFv (Y105G), site-directed mutagenesis was performed by PCR with PrimeSTAR HS DNA polymerase (Takara) using H3K9ac-mintbody and FRET probe expression vectors as templates, and a set of primers (Y105G-for and Y105G-rev).

To obtain stably transfected HeLa cells, PiggyBac transposon vector with EF1α promoter and neomycin resistance (PB533A-2, System Biosciences, Palo Alto, CA, USA) was digested with *Not*I (blunted with T4 DNA polymerase) and *Nhe*I, and inserted with the coding region for Ypet-scFv(195E)-SeCFPdC5H3, which had been prepared by digesting pN2-YPet-scFv(19E5)-SeCFPdC5H3 prepared from *E*. *coli* GM33 (*dam*^-^) by *Xba*I (blunted) and *Nhe*I. *E*. *coli* Top10 (ThermoFisher, Waltham, MA, USA), was used to obtain stable transformants. A control transposon vector for expressing H3K9 mintbody with higher affinity was made similarly using the vector for H3K9v3 tethered to Venus^25^ instead of EGFP^14^.

### Detection of acetylation using fixed U2OS cells

U2OS cells were grown in Dulbecco’s modified Eagle’s medium (DMEM, Wako Pure Chemical Industries, Ltd., Osaka, Japan) supplemented with 10% fetal bovine serum (FBS) and Antibiotic-Antimycotic (Gibco, Thermo Fisher, St. Louis, MO, USA) at 37 °C in 5% CO_2_ with constant humidity. Cells (1 × 10^5^) were seeded in a 24-well plate immersed with a sterilized cover glass (Matsunami Glass, Osaka, Japan). At 90% confluence, the cells were transfected with each plasmid using X-tremeGENE HP DNA transfection reagent (Roche, Indianapolis, IN, USA). Prior to transfection, cells were incubated in 400 μL DMEM without serum or antibiotics. The expression vector (0.3 μg) and 0.9 μL transfection reagent were mixed in 100 μL DMEM at 25 °C for 15 min before being added to the cells. After a 5-h incubation, the medium containing the transfection mixture was replaced with 500 μL DMEM supplemented with 10% FBS. After incubation for 24 h, the cells were either challenged with 150 nM Trichostatin A (TSA, Wako Pure Chemical Industries, Ltd.) dissolved in dimethyl sulfoxide (DMSO) or the same volume of DMSO for 5 h. The cells were washed twice with phosphate-buffered saline (PBS) and incubated with ice-cold methanol for 10 min. After replacing the methanol with PBS, the cover glass was transferred to a 35-mm-diameter glass-bottomed dish (Matsunami Glass) for observation. The differential interference contrast, CFP levels, yellow fluorescent protein (YFP) levels, and FRET images were collected by fluorescence microscopy (IX71, Olympus, Tokyo, Japan) with a 1-s exposure time and a sensitivity gain of four, using the HCImage system equipped with an ImagEM EM-CCD camera (Hamamatsu Photonics, Shizuoka, Japan). The fluorescence intensities were quantified by drawing ROI in each cell containing whole nuclei or cytoplasm region manually.

### Time-lapse monitoring of FRET efficiency

U2OS cells (5 × 10^5^) were seeded in a 35-mm-diameter glass-bottomed dish (Matsunami Glass, Osaka, Japan). At 90% confluence, cells were transfected with each plasmid described above. Fluorescence images were collected every 10 min using a spinning-disk confocal microscope (CSU-W1, Yokogawa Electric, Tokyo, Japan, or Ti-E, Nikon, Tokyo, Japan) with a Plan Apo VC 100X oil-immersion objective lens (NA 1.4) and LU-N4 laser unit (Nikon; 15 mW each at the fiber end), featuring a heat stage (Tokai Hit, Shizuoka, Japan) to keep the culture conditions at 37 °C in 5% CO_2_.

SeCFP and FRET images were obtained by 8% 405 nm laser excitation with 477 nm and 520 nm emission filters, respectively. Fluorescence images of YPet were obtained by 1% 488 nm laser excitation with a 520 nm emission filter. Fluorescence intensities of cytoplasmic and nuclear regions were manually selected as ROIs and quantified by ImageJ (NIH, Bethesda, MD). The fluorescence intensities were quantified by drawing ROI within cytoplasm and nuclei but excluding nucleoli manually. A FRET index was obtained by calculating the fluorescence-intensity ratio (520 nm/447 nm) after background subtraction.

For the observation of HeLa cells, the cells stably expressing H3K9ac-FRET or H3K9ac-Venus probe were established as previously described^26^. Briefly, HeLa cells were transfected with the probe and Tn5-expression plasmids, and 1 mg/mL G418 (Nacalai Tesque, Kyoto, Japan) was added to the culture medium 24 h after transfection. One week later, YFP-positive colonies were isolated under fluorescence microscope. For live-cell imaging, cells were seeded on a 35-mm-diameter glass bottomed dish (MatTek, Ashland, MA) and the medium was replaced with FluoroBrite Dulbecco’s modified Eagle’s medium (Thermo Fisher Scientific) containing supplements before imaging. Fluorescence images were collected using a confocal microscope (FV1000, Olympus) with 458 nm laser excitation, operated by the built-in FV1000 software (FLUOVIEW ver.4.2) and equipped with a PlanApo N 60× Oil SC lens (NA 1.40; Olympus), a heated stage (Tokai Hit), and a CO_2_-control system (Tokken, Kashiwa, Chiba, Japan). The fluorescence intensities were quantified by ImageJ, by drawing ROI within cytoplasm and nuclei but excluding nucleoli manually.

### Immunoblotting

U2OS cells transiently expressing YPet-scFv(19E5)-SeCFPdC5H3 and YPet-scFv(19E5)-SeCFP were grown on a 24-well plate. After incubation with TSA (150 nM for 5 h) or DMSO, cells were washed twice with PBS and lysed with 40 μL 2X Laemmli buffer. Cell lysates were boiled for 5 min and separated by 15% SDS-PAGE before transfer onto polyvinylidene difluoride membranes (Bio-Rad, Hercules, CA, USA) using a semi-dry blotting system (Bio-Rad) at 15 V for 30 min. Membranes were washed three times with 20 mM Tris-HCl (pH 8.0), 150 mM NaCl, and 0.05% Tween 20 (TBST) for 5 min, blocked for 20 min in Blocking-One (Nacalai Tesque), and washed three times for 5 min with TBST. Membranes were then incubated for 1 h at 25 °C with mouse anti-H3K9ac (CMA310^13^; 1 μg/mL), mouse anti-GFP antibody (mFx75, Wako; 1:1000), and mouse anti-actin antibody (Millipore, Danvers, MA, USA; 1:500) in Can-get-signal (Toyobo, Osaka, Japan), washed three times for 10 min with TBST, incubated for 1 h with horseradish peroxidase (HRP)-conjugated rabbit anti-mouse IgG2a (1:2000) in Can-get-signal, and washed three times for 10 min with TBST. Signals were developed using Amersham ECL Prime western-blotting detection reagent (GE Healthcare, Tokyo, Japan) and detected using a luminescent imager LAS-4000 (Fujifilm, Tokyo, Japan).

The stable transfectant HeLa cells treated with either DMSO vehicle or 1 µM TSA for 6 h were lysed and separated by SDS-PAGE as above, before transfer onto a nitrocellulose membrane. The separated membranes were washed thrice with TBST, blocked for 1 h in 1% skim milk in TBST, and incubated for 1 h at 25 °C with either rat anti-H3 (1:20,000) mouse anti-H3K9Ac (1:2000) or mouse anti-GFP antibody (1:100)(Wako) in TBST. The membranes were washed thrice with TBST, and incubated for 30 min at 25 °C with either HRP-goat anti-rat IgG(H + L) (1:5000) (SeraCare, Milford, MA, USA) or HRP-rabbit anti-mouse IgG2a (1:2000) in PBST. Signals were developed as above using a luminescent imager LuminoGraphII (ATTO, Tokyo, Japan).

### Installation of the acetyl-lysine analog into histone H3 at the Lys9 position

To prepare the Lys9 acetylated histone H3 protein, human histone H3.2 containing K9C and C110A mutations was expressed and purified as described previously^27^. To install the acetyl-lysine analog at position 9, Purified H3.2 K9C/C110A protein (3.1 mg) was dissolved in 200 μL of alkylation buffer (0.2 M sodium acetate (pH 4.0), 6 M guanidine−HCl, 7 mM L-glutahione, 50 mM N-vinylacetamide, 100 mM dimethyl sulfide, and 5 mM VA-044 (2,2′-[azobis(dimethylmethlene)]bis(2-imidazoline)dihydrochoride))^28^. After an incubation for 2 h at 70 °C in the dark, the resulting Lys9 acetylated histone H3 proteins were desalted using a PD-10 column (GE Healthcare). The sample was then lyophilized and stored.

### Preparation of polynucleosome

Polynucleosome was prepared essentially as described previously^29^. Briefly, histone octamer was prepared with recombinant human histones H2A, H2B, H3.2(C110A) either with/without K9Ac modification, and H4 by guanidine hydrochloride denaturation and dialysis, followed by the fractionation on a Superdex 200 gel filtration column. The DNA containing twelve tandem repeats of Widom 601 sequence (208 bp) was prepared from the encoding plasmid, and the polynucleosome was reconstituted with the histone octamer by the salt dialysis method. The reconstituted polynucleosome was purified by MgCl_2_ precipitation.

## Supplementary information

Supplementary Video S1

Supplementary Video S2

Supplementary Figures
