# Supplementary material for: Intrabody-based FRET probe to visualize endogenous histone acetylation
Source: Sci Rep. 2019 Jul 15;9:10188. doi: 10.1038/s41598-019-46573-2 (PMC6629662; doi:10.1038/s41598-019-46573-2)
Supplement: Supplementary file 3 — Supplementary Figures [file 41598_2019_46573_MOESM3_ESM.pdf]

Supporting information for

Intrabody-based FRET probe to visualize endogenous histone acetylation

Chan-I Chung[1], Yuko Sato[2], Yuki Ohmuro-Matsuyama[1], Shinichi Machida[3], Hitoshi Kurumizaka[3,$], Hiroshi Kimura[2] & Hiroshi Ueda[1*]

[1] Laboratory for Chemistry and Life Science, and [2] Cell Biology Center, Institute of Innovative Research, Tokyo Institute of Technology, 4259 Nagatsuta-cho, Midori-ku, Yokohama, Kanagawa 226-8503, Japan.

[3] Laboratory of Structural Biology, Graduate School of Advanced Science & Engineering, Waseda University, 2-2 Wakamatsu-cho, Shinjuku-ku, Tokyo, 162-8480, Japan.

[$] Current address: Institute for Quantitative Biosciences, The University of Tokyo, 1-1-1, Yayoi, Bunkyo-ku, Tokyo, 113-0032, Japan.

[*] Corresponding author: H.U. ueda@res.titech.ac.jp

Figure S1. Results of probe screening S-2
Figure S2. The effect of C-terminal H3 tail S-3
Figure S3. Immunoblotting of probe-expressing U2OS cell lysates S-4
Figure S4. FRET probe employing a sensing domain for histone H4K20 monomethylation S-5
Figure S5. Distribution of H3K9ac-mintbody Y105G mutant. S-6
Figure S6. Little FRET with Y105G mutant. S-7
Figure S7. Immunoblotting of probe-expressing HeLa cell lysates. S-8
Figure S8. Preparation of reconstituted polynucleosome S-9

Supplementary videos S1 and S2. Tricostatin A (TSA)-induced accumulation and FRET increase of the probe in the nucleus of U2OS cells.

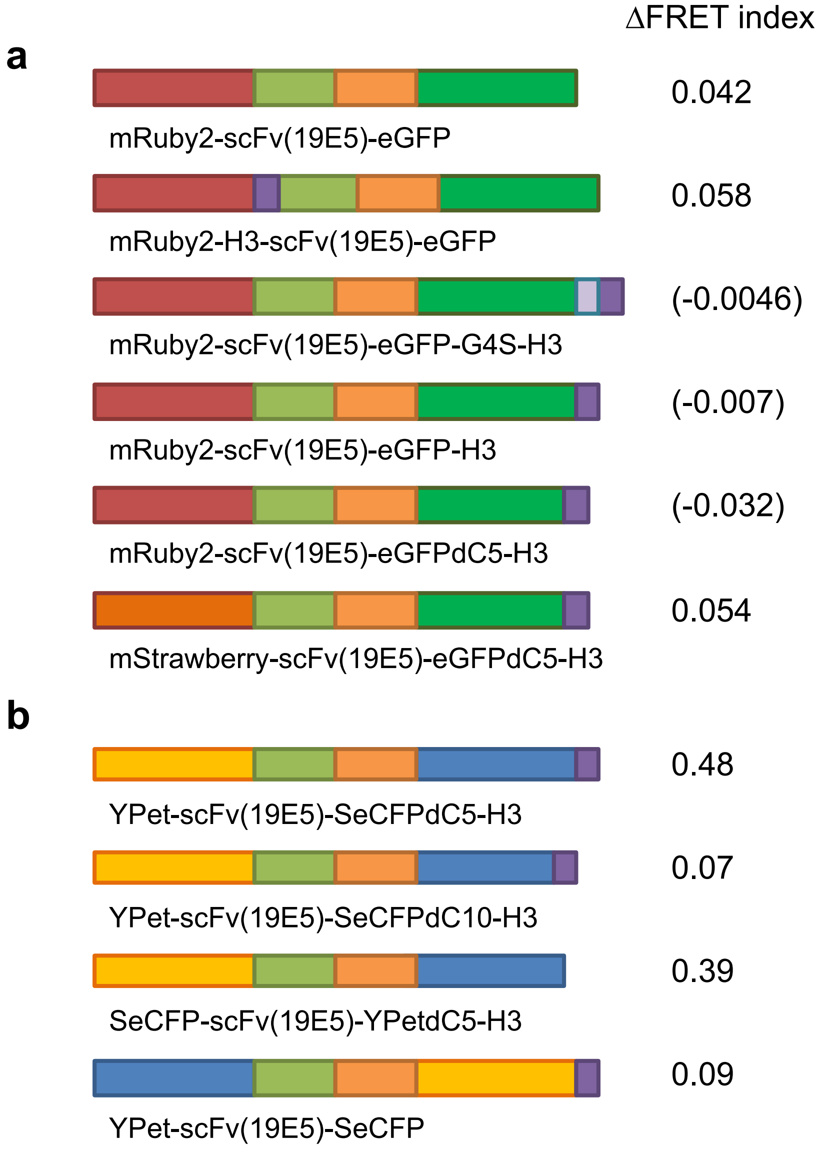

Figure S1. Results of probe screening.
FRET index is defined as the fluorescence intensity ratio of acceptor to donor, which should reflect FRET efficiency. ΔFRET index, which represents the performance of the probe, is the difference of FRET indices between the transiently probe-transfected U2OS cells treated with HDAC inhibitor trichostatin A (1 μM) or dimethylsulfoxide. (a) eGFP-mRuby2 and eGFP-mStrawberry pair probes. (b) SeCFP-YPet pair probes.

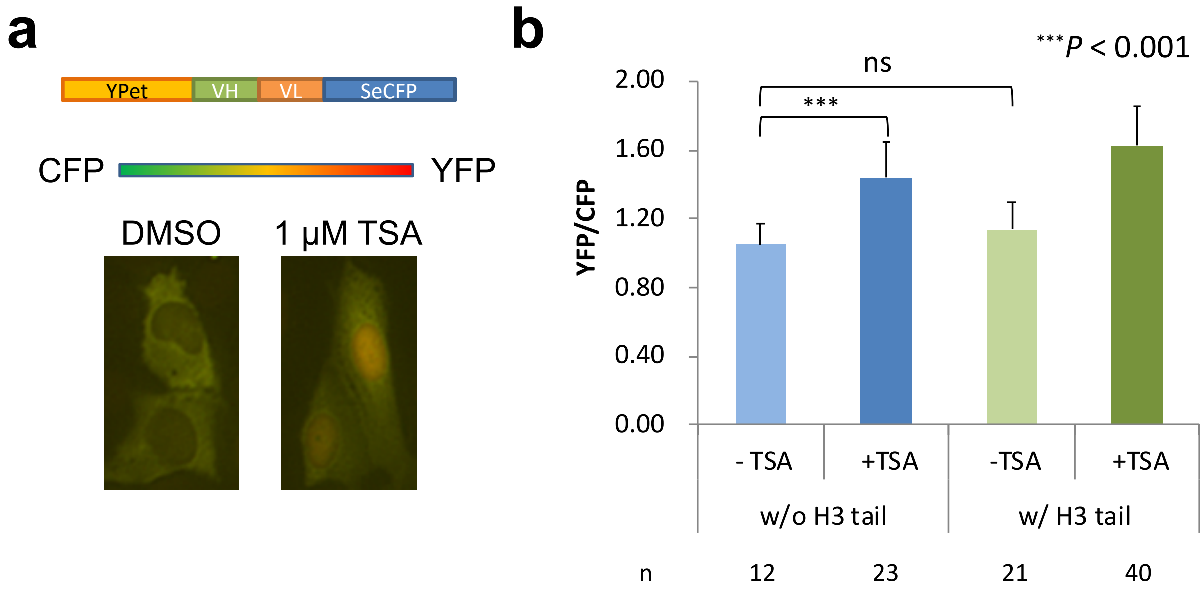

Figure S2. The effect of C-terminal H3 tail.
(a) Pseudocolored FRET image of U2OS cells transiently expressing the Intrabody-based FRET probe without H3 tail.
(b) Ratiometric FRET quantification of fixed cells. (p=0.054 with or without H3 tail in the absence of TSA, $p=9.46\times10^{-4}$ with and without TSA for the probe without H3 tail.)

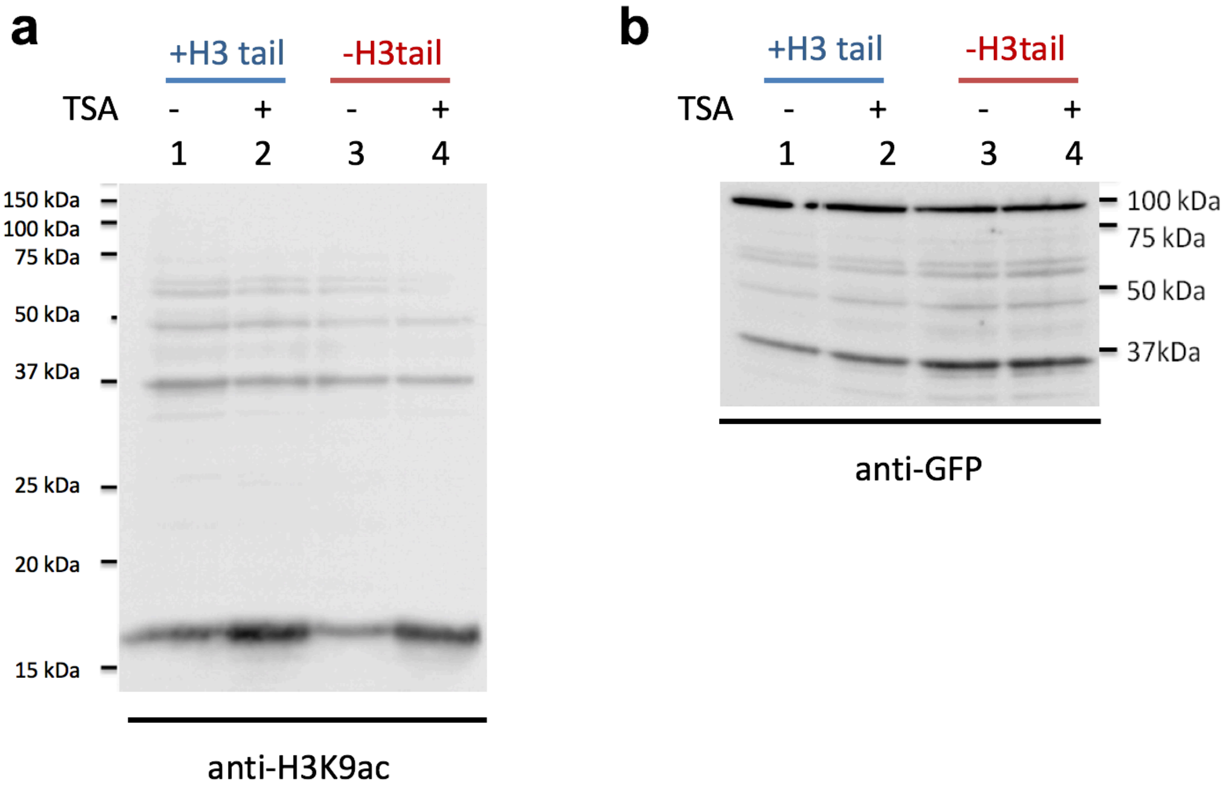

Figure S3. Immunoblotting of probe-expressing U2OS cell lysates. (a) Acetylation levels were analyzed by anti-H3K9ac antibody. The acetylation level of endogenous H3 increased after Trichostatin A (TSA) treatment (Lanes 2 and 4, ~16 kDa). Acetylation of the C-terminal H3 tail in the corresponding probe (Lanes 1 and 2, ~90 kDa) was not detected. (b) The stability and expression levels of the probes were monitored with anti-GFP antibody. Compared with the full-length probes at ~90 kDa, the bands for degraded GFP-containing fragment at 35 kDa are more eminent for the probe without C-terminal H3 tail (Lanes 3 and 4).

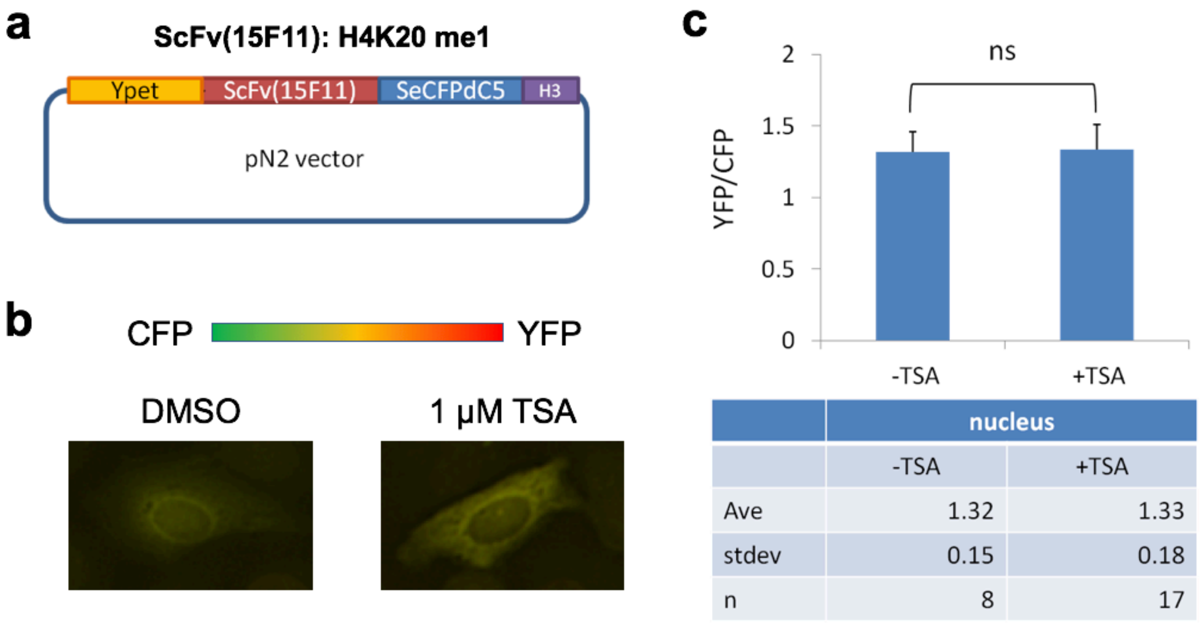

	nucleus	
	-TSA	+TSA
Ave	1.32	1.33
stdev	0.15	0.18
n	8	17

Figure S4. FRET probe employing a sensing domain for histone H4K20 monomethylation.

(a) Schematic presentation of the Intrabody-based FRET probe with sensing domain for histone H4 methylation. YPet : FRET-optimized YFP, SeCFPdC5 : enhanced CFP with 5 aa deletion in C-terminal end, ScFv(15F11) : intrabody of anti H4K40me1, H3 : ARTKQTARKSTGGKAPRKQL.

(b) Pseudocolored FRET image of probe-transfected U2OS cells. There is no specific area which has high FRET efficiency. (c) Ratiometric FRET quantification in nucleus of fixed cells. No significant difference was observed after treatment with HDAC inhibitor ($p > 0.05$).

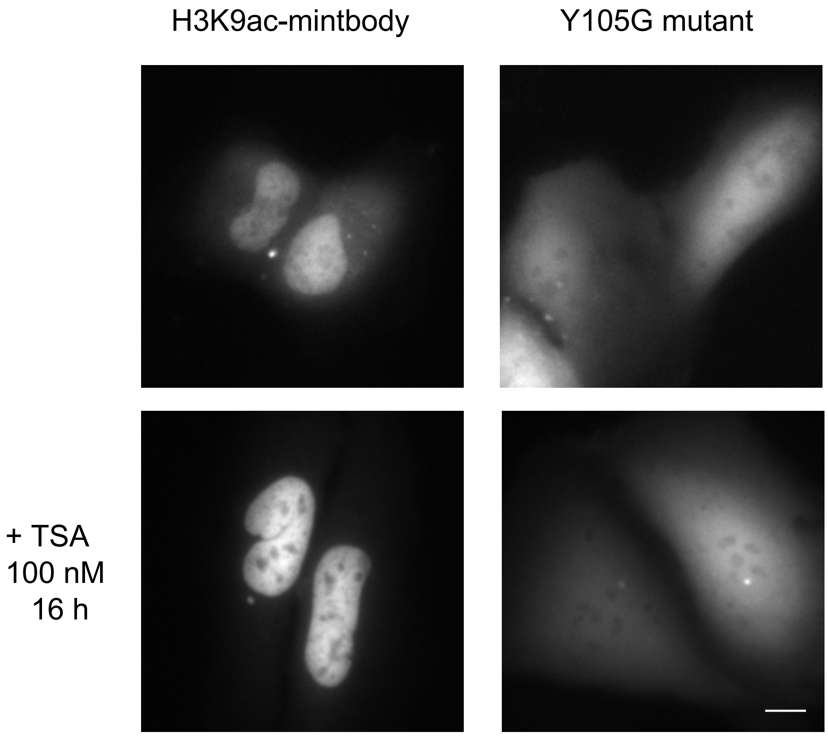

Figure S5. Distribution of H3K9ac-mintbody Y105G mutant. U2OS cells were transfected with H3K9ac-mintbody (wild-type) and Y105G mutant, and fluorescence images without or with TSA were collected. Y105G mutant was less accumulated in the nucleus compared to the wild type even in the presence of TSA. Bar, 10 μm.

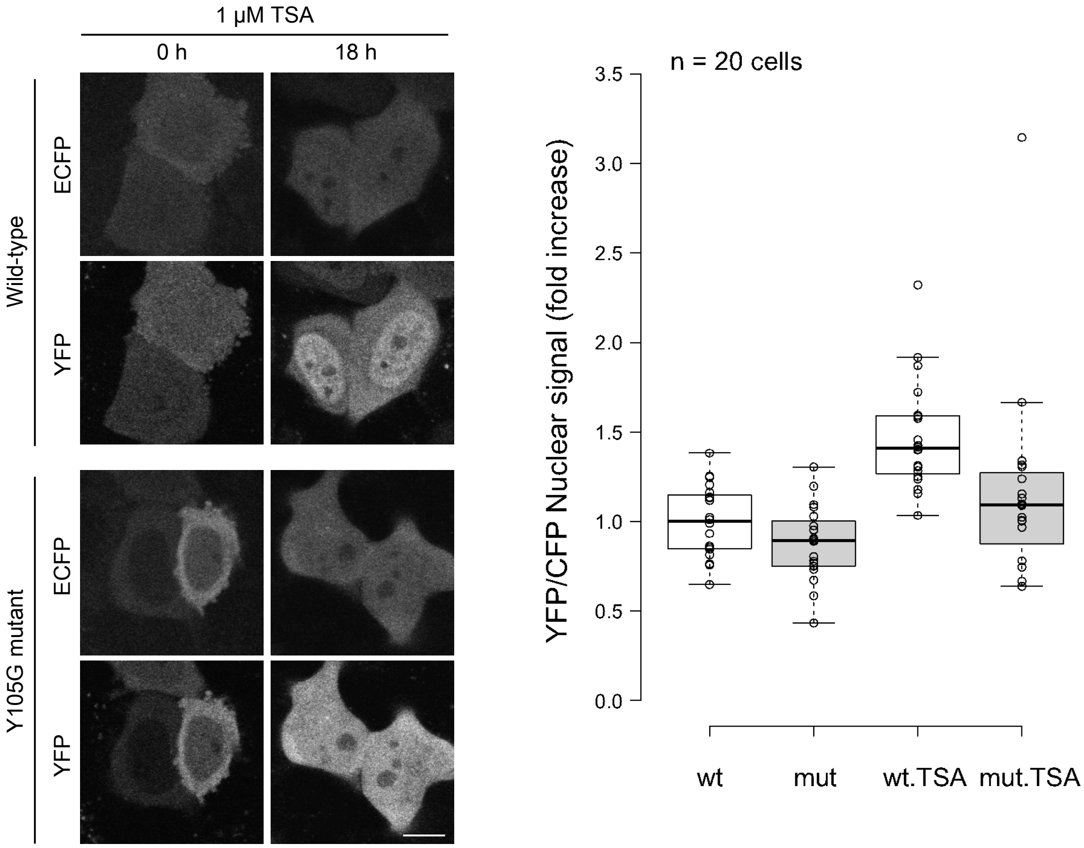

Figure S6. Little FRET with Y105G mutant. HeLa cells were transfected with the FRET probe and Y105G mutant. Confocal images before and after TSA addition were collected (left), and the YFP/CFP ratios in the nucleus were plotted. Y105G mutant showed lower FRET signals than the wild-type without or with TSA. Bar, 10 μm.

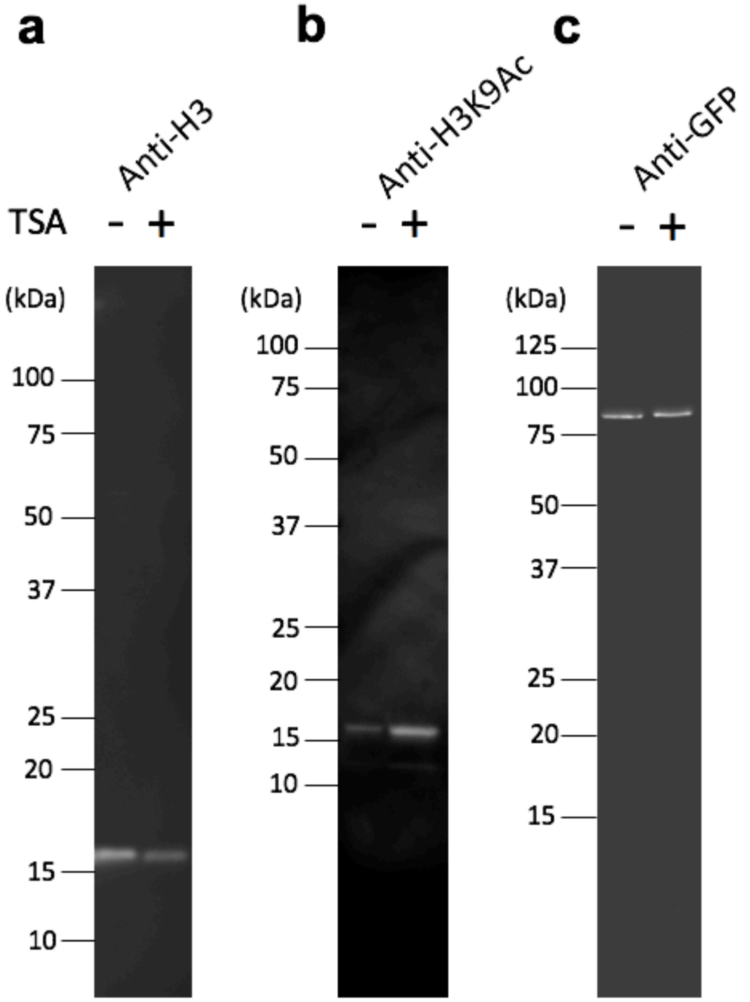

Figure S7. Immunoblotting of YPet-scFv-SeCFPdC5H3 probe-expressing HeLa cell lysates. The amounts of total H3 (a), acetylated H3K9 (b), and the probe (c) in the cells with and without 1 μM TSA treatment were analyzed by anti-H3, anti-H3K9Ac, and anti-GFP antibodies, respectively.

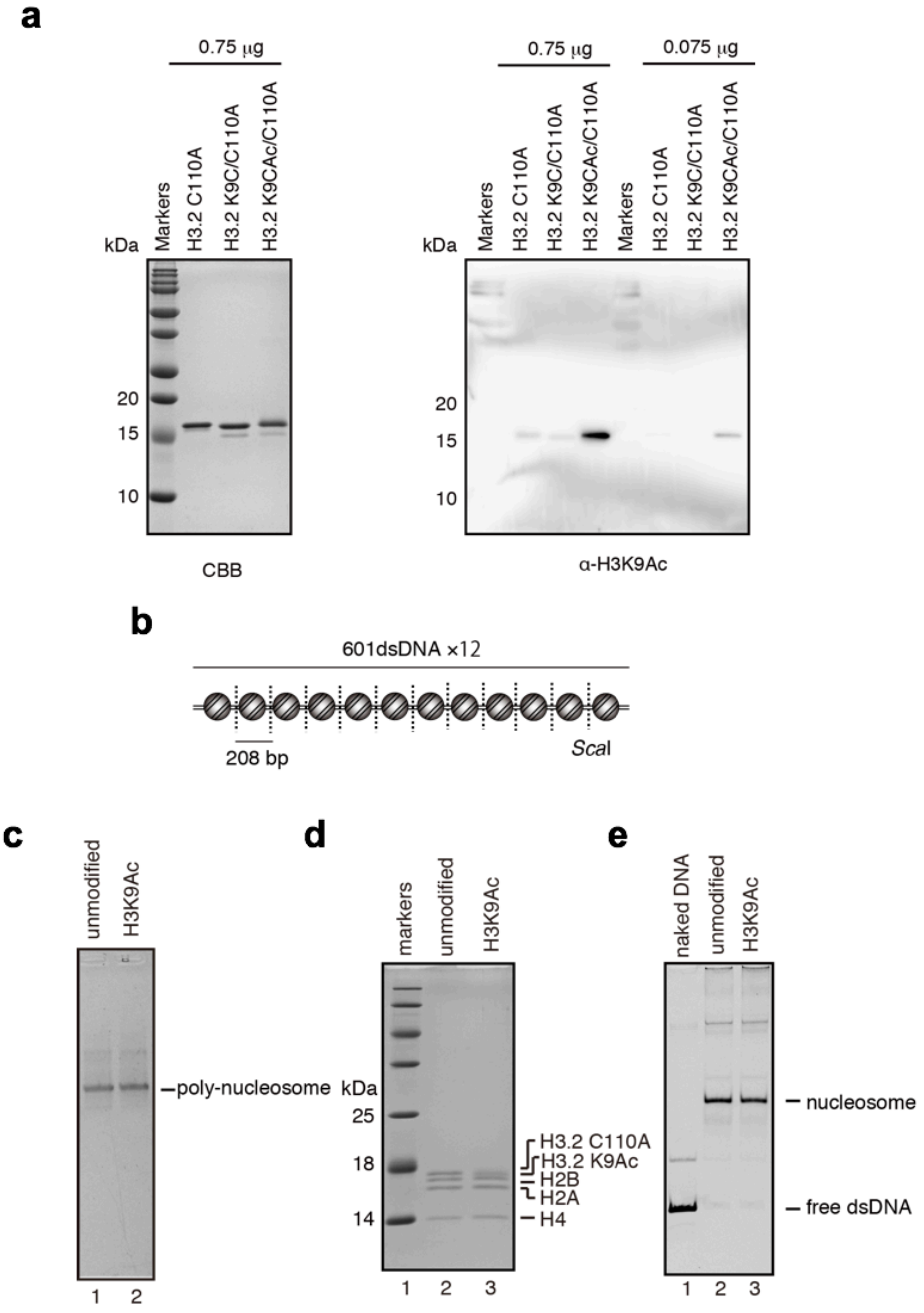

Fig S8. Preparation of reconstituted polynucleosome

(a) Synthesis of H3K9Ac. CBB staining (left) and western blotting (right) of 16% SDS-PAGE showing synthesized H3.2(C110A) with/without acetyl group at Lys9, which was mutated to Cys for chemical modification.

(b-e) Synthesis of polynucleosome. (b) Scheme. (c-e) Evaluation of reconstituted polynucleosome with/without H3K9Ac by gel electrophoresis. (c) A 0.7% agarose-gel stained with ethidium bromide (EtBr). (d) An 18% SDS-polyacrylamide gel stained with CBB. (e) A 5% native polyacrylamide gel stained with EtBr.
